# Identification of Differentially Expressed Genes Involved in the Molecular Mechanism of Pericarp Elongation and Differences in Sucrose and Starch Accumulation between Vegetable and Grain Pea (*Pisum sativum* L.)

**DOI:** 10.3390/ijms20246135

**Published:** 2019-12-05

**Authors:** Pu Yang, Zhonghao Li, Caoyang Wu, Yan Luo, Jing Li, Pengke Wang, Xiaoli Gao, Jinfeng Gao, Baili Feng

**Affiliations:** 1 State Key Laboratory of Crop Stress Biology for Arid Areas, College of Agronomy, Northwest A&F University, Yangling 712100, China; yangpu@nwsuaf.edu.cn (P.Y.); lizhonghao@nwafu.edu.cn (Z.L.); wucaoyang@nwafu.edu.cn (C.W.); 1994lyyl@nwafu.edu.cn (Y.L.); lijing1993@nwafu.edu.cn (J.L.); ylwangpk@163.com (P.W.); gao2123@nwsuaf.edu.cn (X.G.); gjfnxy@nwafu.edu.cn (J.G.); 2 Shaanxi Research Station of Crop Gene Resources and Germplasm Enhancement, Ministry of Agriculture, Yangling 712100, China

**Keywords:** pea (*Pisum sativum* L.), RNA-seq, transcriptome, pod elongation, gene

## Abstract

Pea (*Pisum sativum* L.), as a major source of plant protein, is becoming one of the major cultivated crop species worldwide. In pea, the pericarp is an important determinant of the morphological characteristics and seed yield. To investigate the molecular mechanism of pericarp elongation as well as sucrose and starch accumulation in the pods of different pea cultivars, we performed transcriptomic analysis of the pericarp of two types of pea cultivar (vegetable pea and grain pea) using RNA-seq. A total of 239.44 Gb of clean sequence data were generated, and were aligned to the reference genome of *Pisum sativum* L. In the two samples, 1935 differentially expressed genes (DEGs) were identified. Among these DEGs, three antioxidant enzyme superoxide dismutase (SOD) were detected to have higher expression levels in the grain pea pericarps at the pod-elongating stages. Otherwise, five peroxidase (POD)-encoding genes were detected to have lower expression levels in the vegetative pericarps at the development stage of pea pod growth. Furthermore, genes related to starch and sucrose metabolism in the pea pod, such as *SUS*, *INV*, *FBA*, *TPI*, *ADPase*, *SBE*, *SSS*, and *GBSS*, were found to be differentially expressed. The RNA-seq data were validated through real-time quantitative RT-PCR of 13 randomly selected genes. Our findings provide the gene expression profile of, as well as differential expression information on, the two pea cultivars, which will lay the foundation for further studies on pod development and nutrition accumulation in the pea and provide valuable information for pea cultivar improvement.

## 1. Introduction

Pea (*Pisum sativum* L.), belonging to the legume family, is an annual or biennial crop that originated in the southern part of Europe, the Mediterranean coast, and West Asia [1]. Pea seeds can not only be used as food but also as fodder, and their tender seeds, pods, and seedlings are consumed as edible vegetables. Pea is a widely cultivated crop species and a major source of plant protein for both animal and human consumption [2]. Moreover, the seeds are also used in treating wrinkled skin, diabetes, acne, phlegm, and intestinal inflammation [3,4]. One study suggested that the active ingredients in pea have important anticancer properties [5]. According to the statistics of the Food and Agriculture Organization of the United Nations (FAO), there are 89 countries around the world that plant pea. In 2017, the area for pea cultivation reached 2.669 million hectares, and the total output was 33.29 million tons (FAO) [6].

As a major pulse crop for its protein-rich seeds, much research has focused on the components and development of the seed as well as the breeding of pea. For instance, in some studies on pea seed proteins (*Pisum sativum* L.), researchers found that globulin or storage proteins are the predominant proteins [7,8]. Ronald et al. also isolated and purified two major closely related albumin proteins from pea [9]. Sucrose, starch, alkaloids, galactolipids, trigonelline, piplartine, and essential oils have also been detected in pea seed [4].

In the context of seed development, *ABI5* genes have been found to impact seed maturation and longevity [10]. Enzymes related to glutamine metabolism are also thought to have an impact on the senescence and seed development of pea while antioxidant enzymes regulate pod elongation [11,12]. Currently, studies of simple sequence repeats (SSRs) in the pea genome have gained increasing interest in the field of breeding, with the identification of many novel SSRs that assist in breeding [13,14,15]. While there has been a lot of research on pea seeds and breeding, the studies on peel remain scarce, despite its close ties to seed growth.

Peel is the package or wrapping around the seed, which differentiates and develops from the ovary wall [16]. Some studies have indicated that most peels are likely to have certain characteristics, which allows for their use in different applications, for example, in medicine, as value-added ingredients in various food applications, or as anti-mosquito or deodorant products [17,18]. The pericarp of the pea pod has also shown potential antihyperglycemic activity [19]. Moreover, the pea pericarp is a homologous organ of the leaf, and has a complete photosynthetic function and assists in the growth of seeds but does not play a protective role. Research has indicated that the pea pod has a significant influence in seed photosynthesis in the early stages of pod development and the later stages of plant growth [20]. Thus, the pod weight is the foundation for the formation of multiple seed pods and large pods [20,21]. However, as the edible part of the pea is its seeds, the pericarp is often thrown away as waste, contributing to the lack of research on the pea pod. 

Nowadays, with the development of next-generation technologies, RNA-seq, with its rapidly declining cost and increasing efficiency, is quickly becoming a mainstay in plant genetics and biochemistry research [22,23]. Recently, Iveta and colleagues characterized the differentially expressed genes involved in pea pod dehiscence through combined transcriptomic and metabolomic analyses [24]. While this study provided insights into pod dehiscence, other mechanisms remain unknown, such as those involved in the biosynthesis of components that govern differences in the traits of shape and weight between different pods. In our study, we chose two types of materials (grain pea pod and vegetable pea pod) for transcriptomic analysis to investigate the different regulatory mechanisms of the two different types of pea pod. Our research can provide guidelines for pea breeding and the conversion of byproducts.

## 2. Results

### 2.1. The Phenotypic Traits of Vegetable and Grain Pea Pericarp at Five Developmental Stages

**Figure 1:**
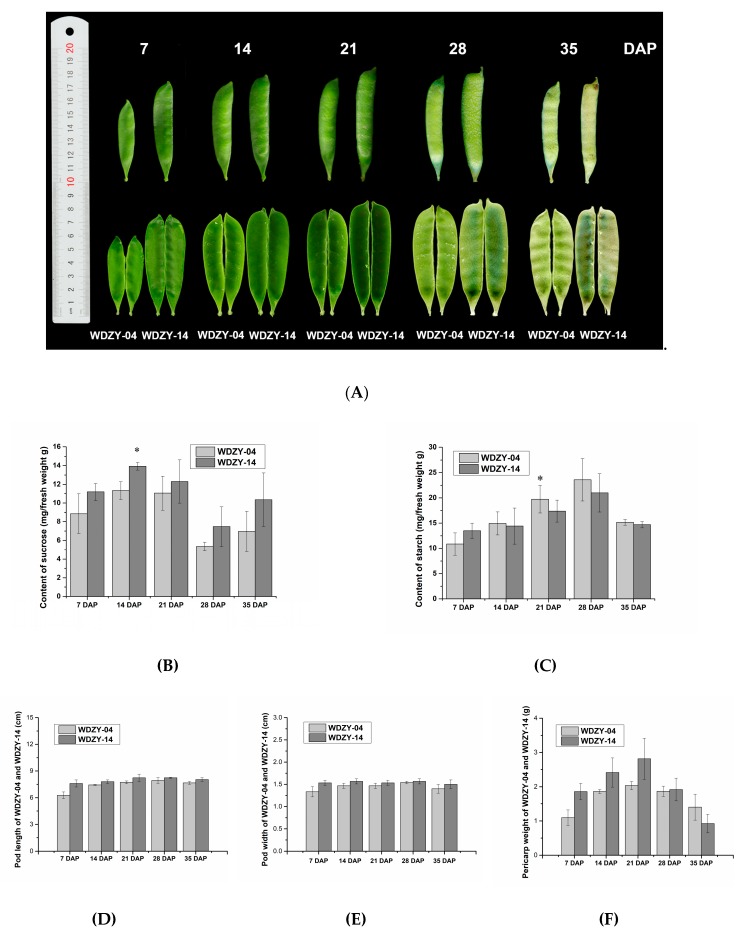
The morphology of the pea pericarp at different developmental stages and pea pericarp size measurement. (**A**) The pod and pericarp of grain pea (WDZY-04) and vegetable pea (WDZY-14) at 7, 14, 21, 28, and 35 days after pollination (DAP). (**B**) Comparison of the sucrose contents in two pea cultivars at five developmental stages. (**C**) Comparison of the starch contents in two pea cultivars at five developmental stages. (**D**–**F**) The length, width, and fresh weight of the pea pericarp at five developmental stages, respectively. The measurement data are shown with standard error bars from three repeated experiments. The data are the means ± SE (*n* = 3). * Denotes a significant difference at *p* < 0.05 (T-test).

Vegetable pea and grain pea show differences in their morphology, nutrition, and taste, with vegetable pea having a higher content of sugar than grain pea, leading to vegetable pea being sweet [25]. In our study, vegetable pea, WDZY-14, and grain pea, WDZY-04, were used. In terms of pod development, the two cultivars showed a significant difference in phenotypic traits. Regarding the pea pericarp, the size in grain pea (WDZY-04) was slightly smaller than in vegetable pea (WDZY-14). The histogram presents the distribution of the shape and weight at each stage (Figure 1D–F). At 7 days after pollination (DAP), the two cultivar pericarps were around 6.3 and 7.6 cm in length, and 1.3 and 1.5 cm in width for the grain pea and vegetable pea, respectively. During pea growth, the size of the pericarp increased, and the largest size was reached at 28 DAP, at which the length and width of the WDZY-04 pea pericarp were 7.9 and 1.54 cm, and those of WDZY-14 were 8.23 and 1.56 cm, respectively. As for the grain pea, WDZY-04, the pericarp size changed most significantly from 7 to 28 DAP, and the length of the pericarp at 28 DAP was almost 1.27 times that at 7 DAP. This result means that, compared with vegetable pea, the pericarp of grain pea elongated more quickly from 7 to 28 DAP (Figure 1D). Meanwhile, during the growth development of the two pea cultivars, the trend of the pericarp weight was increased from 7 to 21 DAP and then decreased. The pericarp weight of WDZY-14 at each stage was heavier than that of WDZY-04 except at 35 DAP. 

The pericarp sucrose contents of the two cultivars differed at each development stage. WDZY-14 accumulated more sucrose than WDZY-04 during growth development (Figure 1B). However, the two cultivars had a similar trend at each development stage, which is that the content of sucrose increased from 7 to 14 DAP, where it reached its peak, followed by a decreasing trend until 28 DAP, whereby it began to increase. Meanwhile, the starch content of the two cultivars presented a single peak pattern during their growth development (Figure 1C). The starch content of the cultivar WDZY-04 was higher than that of WDZY-14.

### 2.2. RNA-seq of the Pea Pericarp Transcriptome

The total RNA was extracted from the pea pericarp of WDZY-14 and WDZY-04 at five growth developmental stages. Then, cDNA libraries were prepared and sequenced using an Illumina HiSeq 4000 platform. We subsequently obtained transcriptomic data from 30 samples that contained the two cultivars of pea pericarp at five stages and their biological replicates. As a result, 239.4 Gb of clean data were produced, and 1641,965,412 clean reads were generated after removing adaptor sequences, ambiguous reads, and low-quality reads (Table 1). The quality of base calling was mostly above Q30, with >96% of the reads having a quality score above Q30. The GC content ranged from 43.05% to 44.16%. The distribution of the base qualities and base percentage composition of the reads of each sample are shown in Figures S1 and S2.

**Figure 2:**
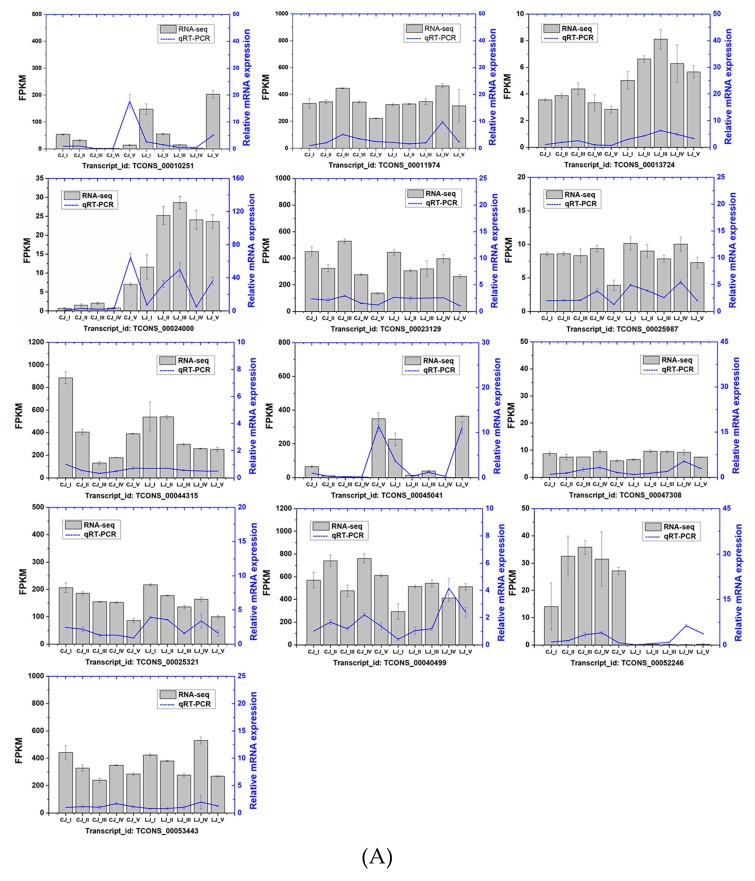

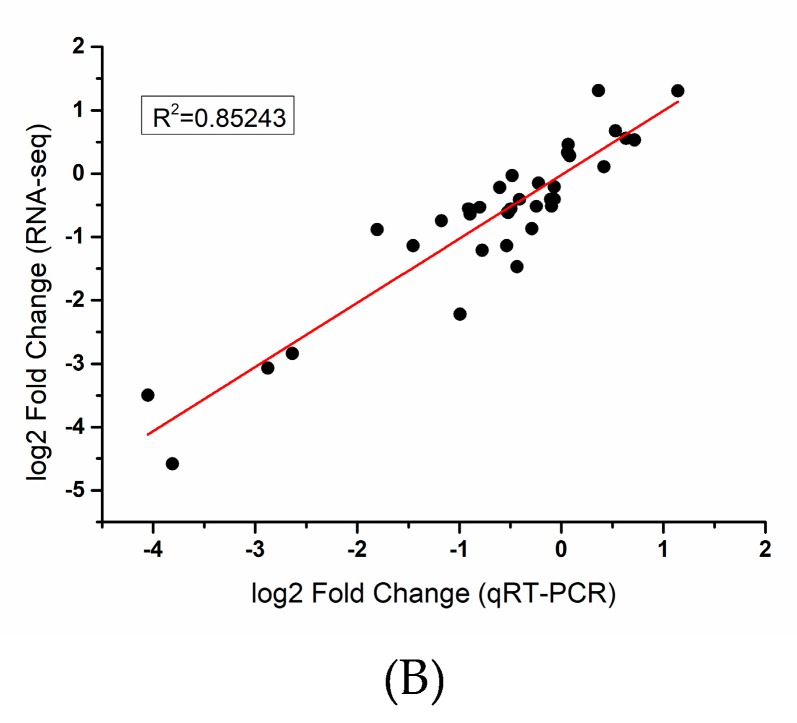
(**A**) qRT-PCR validation of 13 selected genes during the growth development of two pea cultivars. Gray bars indicate the transcript abundance change based on the FPKM values (fragments per kilobase of transcript per million fragments sequenced), according to RNA-seq (left y-axis). Blue lines with standard errors represent the relative expression level, determined by qRT-PCR from independent biological replicates (right y-axis). (**B**) Correlation analysis of 13 selected genes based on qRT-PCR and RNA-seq data; Pearson’s correlation coefficient (r) is 0.92549 (*p* < 0.05).

Moreover, a coverage analysis of the genes and an assessment of the sequencing randomness were conducted in our study to evaluate the quality of the sequencing. Each clean read for each sample was evenly distributed in the gene body (Figure S3). Meanwhile, Pearson’s correlation analysis was performed to evaluate the reproducibility of the biological replicates. As shown in Figure S4, the correlations between samples among the same biological replicates were good, with a value ranging from 0.91 to 1.000. However, the correlations between samples of the biological replicates varied significantly, with a value ranging from 0.04 to 0.88. Meanwhile, qRT-PCR analysis was used to validate the quality of the RNA-seq data. A total of 13 genes were selected. As expected, most of these candidate genes had similar expression tendencies (Figure 2A). While the exact change did not exactly match that of the others, the expression trends of all genes from the qRT-PCR and RNA-seq analyses were largely consistent (Pearson’s correlation coefficient, R^2^ = 0.85) (Figure 2B). The strong correlation between the RNA-seq and qRT-PCR data indicates the reliability of the transcriptomic profiling data.

All of the clean reads were then mapped to the reference genome of *Pisum sativum* Linn (downloaded from NCBI: https://www.ncbi.nlm.nih.gov/nuccore/PUCA000000000.1/), and more than 85% of the clean reads perfectly matched the reference genome (Table 1).

### 2.3. Analysis of the Expression Level and Differentially Expressed Genes (DEGs)

To quantify the expression levels of the transcripts, the Bowtie 2 program was used with RSEM [26,27]. Then, the numbers of mapped reads and the FPKM (fragments per kb per million reads) values were obtained for the following analysis. The statistical results on the expression level (FPKM) of the transcripts for each sample are shown in Table S1. 

**Figure 3:**
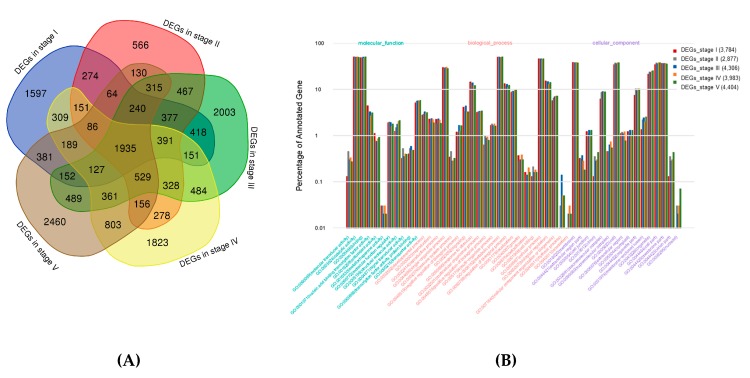
Venn diagram of DEGs and the GO annotation of DEGs in the five compared groups. (**A**) Venn diagram of the number of DEGs in different development stages. (**B**) The GO annotation of DEGs in the different developmental stages.

The genes that were differentially expressed in the vegetable pea pod and grain pea pod at the five growth developmental stages were compared using |log2(fold change)| ≥ 1 and FDR (false discovery rate), with ≤0.05 as a significant cutoff criterion. After data filtering, we detected 6842, 6287, 8767, 8101, and 8417 differentially expressed genes (DEGs) in the two cultivars at the five stages (Table S2). Among these DEGs, 2523, 3036, 3562, 3965, and 3766 were log2 fold change ≥1 (more expressed in the vegetable pea pericarp), and 4319, 3215, 5205, 4136, and 4651 were log2 fold change ≤ −1 (less expressed in the vegetable pea pericarp) (Table S2). A total of 1935 DEGs were common in both cultivars and were putatively considered to be associated with the phenotypic trait differences in this species (Figure 3A). Of the DEGs in the vegetable pea pericarp, 730 out of a total of 1935 were more expressed and 1158 were less expressed in the vegetable pea pericarp.

**Figure 4:**
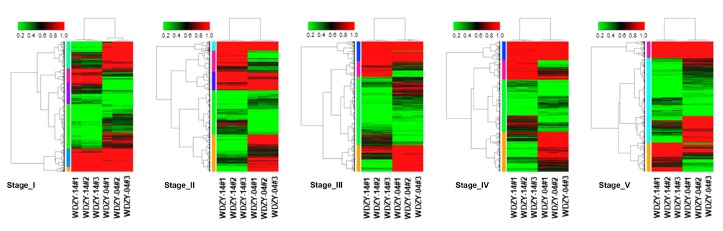
Hierarchical cluster map of differentially expressed genes in the five compared groups. Note: Red indicates high expression and green indicates low expression. The color, from red to green, indicates log10 (FPKM ± 1), from large to small.

In order to understand the expression pattern of DEGs in the five stages for the two cultivars, we conducted hierarchical clustering for the DEGs for the five compared groups (Figure 4). The differentially expressed genes in the five groups were mainly classified into high-expression genes (red) and low-expression genes (green). We grouped genes with a similar expression pattern into a set and used six, five, four, four, and three model profiles to summarize the expression pattern of this subcluster of genes. The DEGs in the five groups fluctuated obviously (more expressed or lower expressed). The expression pattern of the sub-cluster genes in each group is shown in Figure S5.

### 2.4. GO and KEGG Classification

Gene ontology (GO) and Kyoto Encyclopedia of Genes and Genomes (KEGG) enrichment analyses revealed the biological processes, cellular components, molecular functions, and metabolic pathways associated with the identified transcripts from the vegetable pea pericarp and grain pea pericarp. All of the DEGs were divided into three GO categories: Biological process, cellular component, and molecular function (Figure 3B). In the GO annotation, 45, 44, 46, 45, and 45 terms were categorized for the DEGs of developmental stages I–V, respectively. In the biological process, ‘metabolic process’, ‘cellular process’, and ‘single-organism process’ were the most highly represented terms, and ‘membrane part’, ‘membrane’, ‘organelle’, ‘binding’, ‘catalytic activity’, and ‘transporter activity’ were the most enriched in the cellular component and molecular function of the level-two GO term (Figure 3B). 

To investigate the DEG-related pathways, we conducted KEGG annotation of these transcripts. As a result, 19 pathways belonging to 5 categories were retrieved for each compared group. Among them, ‘carbohydrate metabolism’, ‘biosynthesis of other secondary metabolites’, ‘signal transduction’, ‘energy metabolism’, ‘folding, sorting and degradation’, ‘amino acid metabolism’, and ‘translation’ were the top three annotation terms in each group (Figure S6). Similarly, we also analyzed the total DEGs (higher expressed and lower expressed in the vegetable pea pericarp, respectively), and the top three level-two GO terms of more expressed DEGs were ‘biosynthesis of other secondary metabolites’, ‘carbohydrate metabolism’, ‘signal transduction’, ‘folding, sorting and degradation’, ‘amino acid metabolism’, ‘lipid metabolism’, and ‘translation’. The category with the highest number of lower expressed DEGs in the five groups was ‘carbohydrate metabolism’, followed by ‘energy metabolism’, ‘amino acid metabolism’, ‘signal transduction’, ‘translation’, and ‘folding, sorting, and degradation’ (Figure S6).

### 2.5. DEGs Related to Pod Elongation

Reactive oxygen species (ROS) play an important role in such plant functions as cell wall loosening and elongation [28]. The antioxidant enzymes superoxide dismutase (SOD) and peroxidase (POD) were found to be associated with pod growth through the regulation of ROS generation and transformation [12]. In our study, we compared the difference in growth between two cultivars of pericarp and identified the DEGs associated with pericarp elongation. As a result, three superoxide dismutase (SOD)-encoding genes were differentially expressed (Table 2). The gene XLOC_013249 (NR database_id: CAD42655.1), XLOC_030516 (NR database_id: XP_013461079.1, and XLOC_038705 (NR database_id: XP_004508271.1) were more highly expressed in the grain pea pericarp than in vegetable pea pericarp at stages III and V (Table 2). The expression levels of these three genes were not significantly different at stages I and II (supplementary dataset 1). 

For peroxidase (POD), we identified 12 *POD*-encoding genes that included 8 DEGs in vegetable pea and grain pea. Five *POD*-encoding genes, XLOC_016611 (NR database_id: BAD97435.1), XLOC_006267 (NR database_id: BAD97438.1), XLOC_018196 (NR database_id: XP_010104370.1), XLOC_007148, and XLOC_006821 (NR database_id: BAD97436.1), were more highly expressed in grain pea at pod development stages I–V (Table 2). However, the three genes, XLOC_034947 (NR database_id: BAD97436.1), XLOC_037479 (NR database_id: BAD97439.1), and XLOC_031402 (NR database_id: CAA09881.1), exhibited the opposite trend at pod development stage IV, but these three genes were also more highly expressed in grain pea at stages I and III (Table 2).

Moreover, we also analyzed peroxisome metabolism-related genes. The expression levels of the XLOC_010379 (NR database_id: XP_013470503.1) gene encoding peroxisome biogenesis protein were higher in WDZY-04 than in WDZY-14 at stages I and III but lower at stages IV and V. In addition, four genes that participated in the peroxisome pathway (KEGG_id: ko04146) were detected, and it was found that XLOC_019651 (NR database_id: GAU39672.1) was highly expressed in grain pea at stages I–V (Table 2). Instead, the peroxisome biogenesis protein 2 (XLOC_035083, NR_id: XP_004516308.1), peroxisome biogenesis protein 7 (XLOC_036164, NR databse_id: XP_003542988.1), and zinc finger protein-encoding gene (XLOC_036511, NR_id: XP_013444183.1) were more expressed in the vegetable pericarp at development stages I–V (Supplementary Dataset 1).

**Figure 5:**
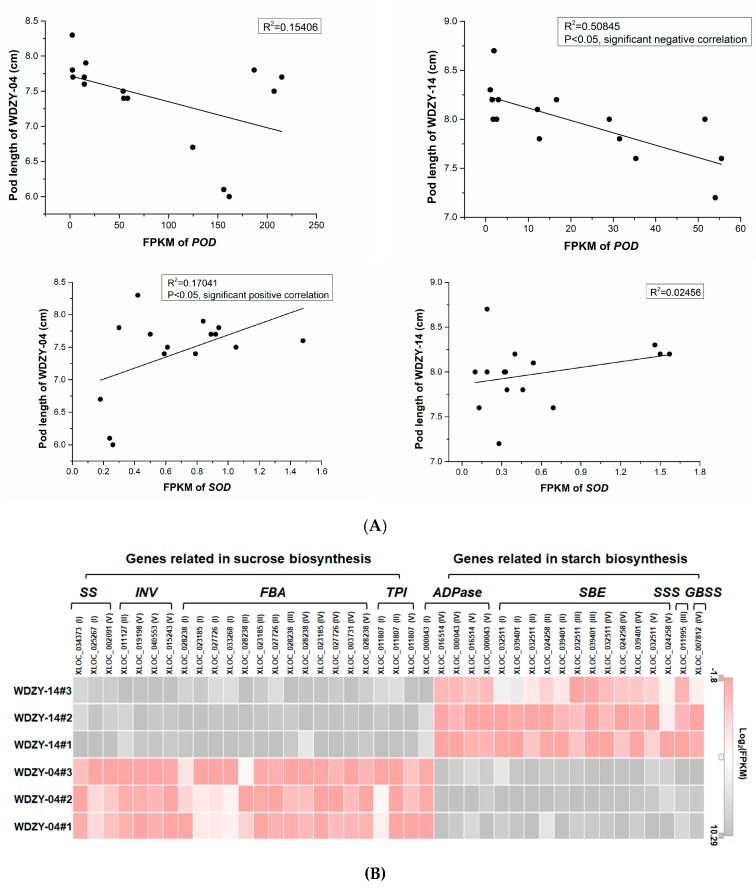
(**A**) The correlations between the expression level of *POD*, *SOD*, and pod length. (**B**) The expression pattern of the key genes involved in sucrose and starch synthesis.

In order to further investigate the relationship between SOD or POD and pod elongation, Pearson’s correlation coefficient analysis was conducted using data of the gene expression level and plant growth. We selected the coefficients related to the phenotypes that met the requirements of statistical significance (multiple testing corrections and an adjusted *p*-value < 0.05). One *SOD*-encoding gene showed a significant positive correlation with pod elongation at WDZY-04, and three *POD*-encoding genes showed a significant negative correlation with pod elongation at WDZY-04 and WDZY-14, respectively (Supplementary dataset 2). To show the correlation clearly, we selected data of one *SOD*-encoding gene (gene_id: XLOC_013249) and one *POD*-encoding gene (gene_id: XLOC_007148) to perform the scatter diagram (Figure 5A). 

### 2.6. DEGs Related to Pod Sucrose Metabolism

Sucrose synthase (SUS) and invertase (INV) play an important role in sucrose metabolism. All of the sugar precursors that participate in sucrose metabolism must be decomposed to hexoses, such as glucose and fructose, or the hexoses (e.g., UDP-glucose) must be ramified by SUS or INV [29]. A total of three encoding sucrose synthase genes were deemed to be the significant DEGs in the two compared groups. The three genes, XLOC_034373 (NR databse_id: O24301.1), XLOC_025267 (NR databse_id: CAC32462.1), and XLOC_002091 (NR databse_id: XP_003591492.2), were more highly expressed in the vegetable pea pericarp at development stages I and V, respectively. The expression level of these three genes in the vegetable pea pericarp were 1.81-, 3.12-, and 1.66-fold higher than that of the grain pea pericarp (Table S3, Figure 5B, Supplementary Dataset 1). 

Invertase-encoding genes were also detected in our study. According to the subcellular localization, the invertase was classified into three types: Cell wall invertase (CWIN), vacuolar invertase (VIN), and cytoplasmic invertase (CIN) [30]. In total, eight invertase-encoding genes were identified, four of which were highly expressed in the vegetable pea pericarp at development stages II and V. The four genes contained one arabinanase/levansucrase/invertase (gene_id: XLOC_011127, NR database_id: XP_003613878.1), one vacuolar acid invertase, PsI-1 (gene_id: XLOC_019198, NR database_id: AAM52062.1), and two cell wall invertases (gene_id: XLOC_040553 and XLOC_015243; NR database_id: AAC17166.1 and CAA84527.1). 

In addition, genes of the soluble sugar metabolism pathway were analyzed. These include fructose-bisphosphate aldolase (*FBA*)- and triosephosphate isomerase (*TPI*)-encoding genes, which participate in fructose and mannose metabolism (ko00051), and the uridylyl transferase-encoding gene, which participates in galactose metabolism (ko00052). Our results showed that five fructose-bisphosphate aldolase-encoding genes, XLOC_028238 (NR database_id: P46257.1), XLOC_023185 (NR database_id: P46256.1), XLOC_003731(NR database_id: Q43088.1), XLOC_027726, and XLOC_033268 (NR database_id: XP_003607065.1), were differentially expressed in the grain pea pericarp and vegetable pea pericarp at various development stages. Among them, one gene, P46257.1 (Transcript_id: XLOC_028238), was more expressed in the vegetable pea pericarp from development stage I to stage V. With plant growth, the expression level of XLOC_028238 decreased form stage I to II, and then increased steadily and reached the second highest at stage III, and then decreased (Supplementary Dataset 1). Similarly, a varying trend of gene XLOC_028238 expression occurred in grain pea, although its FPKM value was lower than that in the vegetable pea pericarp (Table S3, Figure 5B). The other four genes were highly expressed in vegetable pea pericarp at all stages, except for XLOC_027726, which was repressed in stage V. Moreover, one triosephosphate isomerase-encoding gene, XLOC_011807 (NR database_id: XP_013465404.1), was identified, and it was found that this gene was more expressed in the vegetable pea pod at stages I, II, and V but was slightly higher expressed at stages III and IV than the grain pea pod. A total of six uridylyl transferase-encoding genes were detected, but these genes exhibited no great difference in their expression levels in the two compared cultivars.

### 2.7. DEGs Related to Pericarp Starch Synthesis

Starch includes amylose and amylopectin, and granule-bound starch synthase (GBSS), ADP-glucose pyrophosphorylase (ADPase), soluble starch synthase (SSS), starch branching enzyme (SBE), and starch debranching enzyme (isoamylase) are important catalytic enzymes in amylose and amylopectin synthesis [31]. In our study, three *ADPase*-encoding genes, four starch branching enzymes, three *GBSSs*, three phosphoglucomutases, and one soluble starch synthase-encoding gene were identified using RNA-seq (Table S3, Figure 5B, Supplementary Dataset 1). Among these genes, the *ADPase*-encoding gene, XLOC_016514 (NR database_id: CAA65541.1), was significantly higher expressed in the grain pea pericarp than the vegetable pea pericarp at stages IV, where it was 7.19-fold higher in the grain pea pericarp. However, the other *ADPase*-encoding gene, XLOC_000043 (NR database_id: CAA65540.1), was more expressed in the vegetable pea pericarp at stage I (Supplementary Dataset 1). 

Three starch branching enzyme-encoding genes, XLOC_032511, XLOC_039401, and XLOC_024258 (NR_id: Q41058.1), were all highly expressed in the grain pea pericarp and had a low expression in the vegetable pea pericarp from stage I to V. The expression levels of these genes in the grain pea pericarp were 2.80- to 5.14-fold higher than those in the vegetable pea pericarp (Supplementary Dataset 1). In addition, the *GBSS*- and *SSS*-encoding genes were also more highly expressed in the grain pea pericarp at stages III and IV, respectively (Table S3, Figure 5B, Supplementary Dataset 1).

## 3. Discussion

The pea pericarp is an important determinant of the morphological characteristics and seed yield. Mature pericarp usually consists of three parts: The exocarp, mesocarp, and endocarp. As the homologous organ of the leaves, the pod pericarp has a stronger photosynthetic capacity than the leaf at the late stage of seed development, and it can continuously input nutrient products into seeds [32]. The pod has been demonstrated to have a complete functional photosynthetic system, and its contribution to seed yield cannot be ignored [33]. Currently, most studies on the pod have focused on soybeans, beans, and other legumes [34,35]. By contrast, reports on the green pea pericarp are rare as it is generally considered to be a biological waste material. Thus, the pea pericarp remains largely uncharacterized. In our research, we selected two domestic pea cultivars, the vegetable pea cultivar WDZY-14, and the grain pea cultivar WDZY-04, which have significant phenotypic characteristics, to investigate the molecular mechanism of different phenotypic traits. The whole transcriptome data of the two cultivars of pea pericarp at five developmental stages were obtained by high-throughput sequencing technology. About 7.98 Gb of clean reads for each sample were filtered, and approximately 87.78% of the sequences were successfully mapped to the pea reference genome (Table 1). The analysis of the differentially expressed genes reveals that 1935 DEGs co-existed in the five developmental stages. GO and KEGG annotation revealed that these DEGs were mainly involved in the ‘metabolic process’, ‘cellular process’, ‘single-organism process’, ‘membrane part’, ‘membrane’, ‘organelle’, ‘binding’, ‘catalytic activity’, ‘transporter activity’, ‘carbohydrate metabolism’, ‘biosynthesis of other secondary metabolites’, ‘signal transduction’, ‘energy metabolism’, ‘folding, sorting, and degradation’, ‘amino acid metabolism’, and ‘translation’.

Reactive oxygen species (ROS) can impact metabolism and plant growth by interacting with proteins, carbohydrates, and other components in the cell and play an important role in cell wall loosening and elongation [36]. Research into the pericarp elongation of *Pisum sativum* suggested that high levels of O_2_^−^ and ·OH may have an impact on cell wall loosening and cell elongation, and that superoxide dismutase (SOD) and peroxidase (POD) were associated with pericarp growth through the regulation of ROS generation and transformation [28]. A high SOD activity and low-level POD can increase pod wall thickness by regulating ROS [12]. In our study, we identified three *SOD*-encoding genes that were differentially expressed in the two cultivars. More *SOD*-encoding genes were expressed in the grain pea pericarp at development stages III and IV. According to our measurements, the size of the grain pea WDZY-04 pericarp experienced the greatest change from stage I to stage IV, especially in the early developmental stages. Thus, we suspect that more *SOD*-encoding genes in the pod of grain pea contributed to the faster pod elongation. In addition, five *POD*-encoding genes were lower expressed in the vegetable pea pericarp at the developmental stage. A previous study indicated a positive relation between pod wall thickness and SOD activity and a negative relation between pod wall thickness and POD activity, and these two enzymes are synergic and responsible for pod growth through the regulation of ROS generation [12]. Similar to the findings of Liu et al. [12], our Pearson’s correlation coefficient analysis also showed a significant positive correlation between the expression level of one *SOD*-encoding gene and pod elongation at WDZY-04 and a significant negative correlation between the expression level of three POD-encoding genes at WDZY-04 and WDZY-14. Thus, we think that the more *POD*-encoding genes found in the grain pea pod is one of the reasons for the vegetable pea pod being longer than the grain pea pod. These results further suggest that POD may have contributed to pod elongation. 

Vegetable pea typically has a higher content of sugar than grain pea, so it is sweeter than grain pea [25]. This remarkable phenotype characteristic has led to the division of the food-oriented pea into two types (vegetable pea and grain pea). Pea pericarp consists of wrapped seeds that are directly linked to each other, so it plays an important role in both pea seed yield and quality. The reason for peas being sweeter is closely related to sucrose metabolism in plant cells. The distribution of sucrose to storage organs, such as seeds, fruits, and tubers, is one of the most important factors determining crop yield and quality, so sucrose metabolism also plays a key role in plant growth and development [37,38]. After unloading the phloem into sinks, sucrose must be degraded into hexoses or their derivates to become metabolically available [39]. Sucrose synthase (SUS) and invertase (INV) play an important role in this process. Sucrose synthase is a glycosyltransferase, and can convert sucrose into UDP-glucose and fructose in the presence of UDP [40,41]. Invertase is a hydrolytic enzyme that hydrolyzes sucrose into glucose and fructose [42]. In our study, we identified three sucrose synthase-encoding genes that were more highly expressed in the vegetable pea pericarp than in grain pea, and hypothesized that these three encoding genes are involved in sugar synthesis in pea pericarp as one of the molecular mechanisms that contribute to the sweet taste of peas. Meanwhile, we also identified four invertase-encoding genes that were more highly expressed in the vegetable pea pericarp, and suggested that these genes may be one of the factors responsible for the higher sweetness of the vegetable pea pericarp. Furthermore, the other genes related to sucrose metabolism were also analyzed. Uridylyl transferase, fructose-bisphosphate aldolase, and triosephosphate isomerase were all more highly expressed in the vegetable pea pericarp than in the grain pea cultivar. These results suggested that these genes play a role in the sweet phenotype. At the same, the identified expression of these genes, associated with sucrose metabolism in the pea pericarp, was consistent with previous studies on pea seeds [25]. We also hypothesized that the traits of the pea pericarp could also affect the sweet traits of pea seeds.

Similarly, considering the difference in starch content between the two peas, we identified the DEGs related to starch metabolism in the pea pericarp to investigate the starch synthesis mechanism in the pea pericarp and the relation between pea and the pea pericarp. Starch is the main carbohydrate that accumulates in mature seeds. Genes involved in starch biosynthesis have been reported in a previous study, and include *SUS*, *GBSS*, *SS*, *BE*, *ADPase*, and *DBE* [31]. However, pea pericarp and pea starch biosynthesis remain largely unknown. We characterized the expression levels of *ADPase*, *GBSS*, the starch branching enzyme phosphoglucomutase, and soluble starch synthase-encoding genes, and the results revealed that most of these genes related to starch synthesis were highly expressed in the grain pea pericarp, especially at the later stage of plant growth. At the later stage of pea seed development, the grain pea seed accumulated a lot of starch and its starch content was higher than that of the vegetable pea. Interestingly, we observed that the starch-related genes in the grain pea pericarp were more highly expressed at a later development stage compared to vegetable pea, and we also presumed that the mechanism of pea pericarp starch metabolism may affect pea seed starch accumulation. 

In short, our study determined the phenotype characteristics of two pea pericarps, and a transcriptomic analysis was conducted. The aim was to research the molecular mechanisms involved in different traits of the vegetable pea pericarp and grain pea pericarp. Numerous DEGs were identified as being involved in ROS generation and sucrose and starch biosynthesis in the pea pericarp. Taken together, these results may facilitate the understanding of the molecular mechanisms involved in the sweetness and growth differences between the two types of peas, as well as aid in the construction of a genetic map for pea.

## 4. Materials and Methods 

### 4.1. Plant Material

The domesticated pea (*Pisum sativum* L.) samples, including the vegetable pea cultivar “WDZY-14” and the grain pea cultivar “WDZY-04”, were planted in an experimental farm at Northwest A&F University, Yangling (E108°4′/N34°16′/511 m), Shaanxi, China, in the 2018 growing season. Three biological replicates were set up for each cultivar. The pea is a self-pollination species, and the first day of the flower being fully open is defined as the first day of fertilization (0 DAP) [43]. After pollination on 17 April (the flower fully open, 0 DAP), we collected pea pericarp at five developmental stages according to the pea growth of the two cultivars for further study: Stage I (7 days after pollination, 7 DAP), stage II (14 days after pollination, 14 DAP), stage III (21 days after pollination, 21 DAP), stage IV (28 days after pollination, 28 DAP), and stage V (35 days after pollination, 35 DAP). Each pericarp was considered one biological replicate, and we collected three pericarps in three plants at five developmental stages. Thus, 30 samples were collected in total (two cultivars at five developmental stages, with three replicates, e.g., the number, WDZY-04-I#1, was the cultivar, WDZY-04, from the first replicate at stage I). Figure 1A shows the different developmental stages of the pericarp in the two pea cultivars. After harvest, the tissues were immediately frozen in liquid nitrogen and stored at −80 °C until required. For RNA extraction, we divided the pericarp and seed of the pea of each cultivar. 

### 4.2. Measurement of the Phenotypic Traits of the Two Cultivars

An estimation of the sugars and starches was carried out on the whole pericarps of the two cultivars harvested at each developmental stage according to a previous methodology [44]. A total of 0.1 g (dry weight) of the samples were ground into fine powder and placed into a 10-mL centrifuge tube, and then homogenized in 4.0 mL of ethanol (80%). After extraction in a water bath (80 °C) for 40 min and centrifugation, the supernatant was collected, and 4.0 mL of 80% ethanol were added to the precipitate, for another extraction. After decolorization in an 80 °C water bath for 30 min, the supernatants were prepared to a constant volume of 25 mL. After filtration, the filtrate was taken for analysis. The soluble total sugar content and starch content were determined by the anthrone method, and the sucrose content was determined by the resorcinol method.

### 4.3. Illumina Sequencing and Mapping

The total pea pericarp RNA was extracted using an RNAprep Pure Plant kit (Bio TeKe, Beijing, China) following the manufacturer’s instructions. The purity and concentration of each RNA sample was determined using a NanoDrop 2000 spectrophotometer (Thermo Scientific, Wilmington, DE, USA). The equal amounts of RNA from each sample were pooled for cDNA library construction. Stranded cDNA libraries were constructed using a NEBNext Ultra Directional RNA Library Prep Kit (cat#E7420, NEB, UK) according to the manufacturer’s protocols. Briefly, the mRNA was fragmented into 250 to 450 bp followed by first strand cDNA synthesizing. Then, dUTP was added as a marker during the syntheses of the second strand cDNA. Finally, the double strand cDNA was digested with Uracil - DNA Glycocasylase (UDG) before PCR reaction. In this way, only the first strands of cDNA were kept and sequenced. Transcriptome sequencing was carried out on the Hiseq 4000 (Illumina, San Diego, CA, USA) platform using a paired-end run (2 × 150bp).

After removing the adaptor sequences and low-quality sequences (Q < 30), the clean reads were aligned to the reference genome sequences of the *Pisum sativum* Linn genome (downloaded from NCBI: https://www.ncbi.nlm.nih.gov/nuccore/PUCA000000000.1/) using Tophat2 software with default parameters [45]. All of the raw data, generated by sequencing, were deposited in NCBI SRA under the following accession: PRJNA548433.

### 4.4. Expression Level Analysis and Gene Annotation

A fragments per kilobase per million (FPKM) analysis, which simultaneously considers the sequencing depth and length of a count, was used to measure the gene expression levels [46]. Genes with an expression level of at least 1 FPKM in at least one sample were retained after removing genes with low expression levels. The differentially expressed genes (DEGs) were identified by deseq2 in R software, with an FDR (false discovery rate) ≤ 0.05 and |log2 (fold change)| ≥1 between two samples. 

Gene ontology (GO) enrichment analysis and normalized gene expression data were used to identify the function of and relationships between differentially expressed genes. The identified DEGs were subjected to GO (Gene Ontology: http://geneontology.org/) and KEGG pathway enrichment analysis using phyper in R software. Moreover, the NR (NCBI nonredundant protein sequences (https://www.ncbi.nlm.nih.gov/refseq/about/nonredundantproteins/) and GO and KO (KEGG ORTHOLOGY, https://www.kegg.jp/kegg/ko.html)) annotation of transcripts were carried out using BLAST (cutoff *E*-value < 1 × 10^−5^).

### 4.5. qRT-PCR Validation

A total of 13 genes were selected to validate the RNA-seq data. The HiScript^®^ II Q RT SuperMix for qPCR (+g DNA wiper) Kit (Nanjing, China) was used according to the manufacturer’s instructions to generate the first cDNA after extracting the total RNA from six samples subjected to RNA-seq. The gene-specific primers were produced by Primer 5.0 software (Premier, Canada), and the primer sequence is listed in Table S4. The gene-specific primer was synthesized by Sangon Biotech Co., Ltd. (Shanghai, China). ChamQTM SYBR^®^ Color qPCR Master Mix (10 μL; Vazyme, Nanjing, China) was mixed with gene-specific primers, sterilized water, and the synthesized cDNA, with 20 μL as the total reaction volume. The reaction were performed on an qTOWER 2.2 (Analytik Jena AG, Jena, Germany). The two-step quantitative RT-PCR program began at 95 °C for 30 s, followed by 40 cycles of 95 °C for 10 s and 60 °C for 30 s. Each reaction was carried out with three biological replicates and three technical replicates. Tubulin was used as the internal reference gene. The data were analyzed using the 2^−ΔΔ*C*t^ method to obtain relative mRNA expression data.

## 5. Conclusions

We investigated the molecular mechanism responsible for the phenotypic traits of the pericarp of two pea cultivars through a transcript analysis of five developmental stages. We analyzed the DEGs of the two cultivars and determined their relative expression levels. Then, hierarchical clustering was used to analyze the expression pattern of the DEGs. As a result, we identified 1935 DEGs common to the five developmental stages of pea. Moreover, we identified important genes related to pod elongation and starch and sucrose synthase that may influence the seed quality. Our research will provide a basis for further studies on starch biosynthesis in pea and a reference for heredity breeding.

## Figures and Tables

**Table 1:** Statistical analysis of the pea pericarp transcriptome data of vegetable pea (WDZY-14) and grain pea (WDZY-04) at five growth developmental stages.

Transcriptome Sample	Total Clean Reads	Clean Bases (bp)	Q30 (%)	GC (%)	Total Mapped Reads (%)
WDZY-04_ I#1	50,177,200	7,327,792,349	96.765	43.575	44,475,396 (88.6%)
WDZY-04_ I#2	49,061,332	7,188,004,754	96.825	43.245	43,387,875 (88.4%)
WDZY-04_ I#3	50,406,494	7,306,478,571	96.675	43.37	44,730,739 (88.7%)
WDZY-04_ II#1	52,539,172	7,662,278,764	96.74	43.3	46,613,759 (88.7%)
WDZY-04_ II#2	43,217,524	6,246,391,018	96.585	43.475	38,215,715 (88.4%)
WDZY-04_ II#3	64,372,148	9,362,931,919	96.835	43.36	57,145,999 (88.8%)
WDZY-04_ III#1	70,119,664	10,202,409,573	96.815	43.245	62,179,404 (88.7%)
WDZY-04_ III#2	42,849,086	6,201,019,405	96.655	43.22	37,836,945 (88.3%)
WDZY-04_ III#3	50,627,610	7,391,282,480	97.485	43.045	45,179,447 (89.2%)
WDZY-04_ IV#1	46,268,006	6,789,317,950	96.42	43.15	40,851,769 (88.3%)
WDZY-04_ IV#2	55,534,482	8,050,533,372	96.705	43.16	49,204,948 (88.6%)
WDZY-04_ IV#3	44,742,284	6,511,953,243	97.725	43.335	39,799,834 (89.0%)
WDZY-04_ V#1	51,782,254	7,463,115,630	96.84	43.2	44,300,719 (85.6%)
WDZY-04_ V#2	64,122,736	9,251,735,817	96.825	43.435	54,682,602 (85.3%)
WDZY-04_ V#3	51,512,076	7,460,292,563	96.78	43.405	43,870,727 (85.2%)
WDZY-14_ I#1	53,175,366	7,763,234,698	97.45	43.96	48,054,402 (90.4%)
WDZY-14_ I#2	46,601,580	6,807,140,735	97.435	43.93	42,061,045 (90.3%)
WDZY-14_ I#3	48,438,836	7,005,642,104	97.1	43.855	43,603,612 (90.0%)
WDZY-14_ II#1	48,696,214	7,105,553,520	97.425	43.59	43,878,335 (90.1%)
WDZY-14_ II#2	88,115,270	12,807,329,980	97.37	43.63	79,369,026 (90.1%)
WDZY-14_ II#3	47,762,154	6,956,660,221	97.43	43.425	43,127,846 (90.3%)
WDZY-14_ III#1	46,007,202	6,562,236,455	97.19	43.565	40,383,101 (87.8%)
WDZY-14_ III#2	77,181,058	11,150,851,050	97.51	43.715	68,087,616 (88.2%)
WDZY-14_ III#3	73,287,766	10,600,678,854	97.46	43.68	64,597,938 (88.1%)
WDZY-14_ IV#1	46,437,750	6,721,682,138	96.93	43.925	41,388,924 (89.1%)
WDZY-14_ IV#2	52,647,274	7,628,273,045	97.44	43.43	47,093,257 (89.5%)
WDZY-14_ IV#3	56,984,532	8,236,432,557	96.845	43.255	50,542,076 (88.7%)
WDZY-14_ V#1	58,306,756	8,466,331,576	97.485	44.04	46,961,785 (80.5%)
WDZY-14_ V#2	45,359,128	6,567,548,081	97.49	44.155	36,311,644 (80.1%)
WDZY-14_ V#3	65,632,458	9,522,635,831	97.47	44.01	52,782,939 (80.4%)

**Table 2:** DEGs and enzymes involved in pod elongation.

Function	Gene_ID (NR database _id)	Growth Stage	WDZY -04#1 (FPKM)	WDZY -04#2 (FPKM)	WDZY -04#3 (FPKM)	WDZY -14#1 (FPKM)	WDZY -14#2 (FPKM)	WDZY -14#3 (FPKM)
***SOD* (superoxide dismutase)**	XLOC_013249 (CAD42655.1)	Stage III	0.84	0.92	1.48	0.32	0.19	0.19
	XLOC_030516 (XP_013461079.1)	Stage V	6.89	4.96	5.59	2.06	1.56	2.14
	XLOC_038705 (XP_004508271.1)	Stage V	7.67	7.41	7.77	2.66	2.17	2.11
***POD*** **(peroxidase)**	XLOC_016611 (BAD97435.1)	Stage I	12.48	14.26	7.99	0.67	0.77	0.52
	XLOC_007148 (BAD97436.1)	Stage I	156.02	161.58	124.67	51.6	55.49	54.04
	XLOC_034947 (BAD97436.1)	Stage I	99.49	99.11	82.03	4.86	5.75	5.72
	XLOC_037479 (BAD97439.1)	Stage I	38.62	37.61	37.81	10.94	12.67	15.38
	XLOC_031402 (CAA09881.1)	Stage I	259.53	233.79	187.8	60.94	63.29	70.8
	XLOC_006267 (BAD97438.1)	Stage I	44.78	41.83	36.77	14.98	15.67	15.38
	XLOC_006821 (BAD97436.1)	Stage I	2.94	1.66	1.73	0	0.18	0
	XLOC_006267 (BAD97438.1)	Stage II	14.48	14.26	13.78	5.87	6.66	6.89
	XLOC_016611 (BAD97435.1)	Stage II	27.55	22.79	25.32	1.18	1.55	1.83
	XLOC_016611 (BAD97435.1)	Stage III	26.89	28.71	30.24	2.3	2.01	1.81
	XLOC_018196 (XP_010104370.1)	Stage III	0.28	0.07	0.32	0	0	0
	XLOC_007148 (BAD97436.1)	Stage III	16.03	14.61	14.63	2.5	1.93	1.74
	XLOC_034947 (BAD97436.1)	Stage III	1.18	1.42	1.45	0.38	0.09	0.15
	XLOC_031402 (CAA09881.1)	Stage III	40.27	39.64	36.16	6.84	6.46	5.4
	XLOC_006267 (BAD97438.1)	Stage III	27.95	26.15	25.67	7.26	5.53	5.6
	XLOC_016611 (BAD97435.1)	Stage IV	26.56	24.06	21.55	0.91	0.69	0.74
	XLOC_034947 (BAD97436.1)	Stage IV	0.27	0.16	0.16	2.38	3.29	3.73
	XLOC_037479 (BAD97439.1)	Stage IV	0.43	1	0.48	1.61	1.62	1.81
	XLOC_031402 (CAA09881.1)	Stage IV	0.62	0.99	0.89	2.57	2.7	4.32
	XLOC_007148 (BAD97436.1)	Stage V	186.77	214.85	207.05	16.61	12.58	12.14
**peroxisome**	XLOC_010379 (XP_013470503.1)	Stage I	0.8	1.2	1.46	0.17	0.24	0.5
	XLOC_010379 (XP_013470503.1)	Stage III	1.48	1.55	1.92	0.33	0.47	0.51
	XLOC_010379 (XP_013470503.1)	Stage IV	0.74	0.96	1.06	1.93	2.08	2.06
	XLOC_010379 (XP_013470503.1)	Stage V	0.49	0.33	0.28	0.77	0.55	0.72
**ko(04146)**	XLOC_019651 (GAU39672.1)	Stage I	71.72	61.62	56.4	0	0	0
	XLOC_019651 (GAU39672.1)	Stage II	36.91	42.82	46.38	0	0	0
	XLOC_019651 (GAU39672.1)	Stage III	50.38	54.52	25.55	0	0	0
	XLOC_019651 (GAU39672.1)	Stage IV	27.28	15.42	23.63	0	0	0
	XLOC_019651 (GAU39672.1)	Stage V	38.71	26.16	19.8	0	0	0

## Supplementary Materials

Supplementary materials can be found at https://www.mdpi.com/1422-0067/20/24/6135/s1.
